# Exceptionally active iridium evolved from a pseudo-cubic perovskite for oxygen evolution in acid

**DOI:** 10.1038/s41467-019-08532-3

**Published:** 2019-02-04

**Authors:** Yubo Chen, Haiyan Li, Jingxian Wang, Yonghua Du, Shibo Xi, Yuanmiao Sun, Matthew Sherburne, Joel W. Ager, Adrian C. Fisher, Zhichuan J. Xu

**Affiliations:** 1 School of Material Science and Engineering, Nanyang Technological University, 50 Nanyang Avenue, Singapore, 639798 Singapore; 2 The Cambridge Centre for Advanced Research and Education in Singapore, 1 CREATE Way, Singapore, 138602 Singapore; 3 Solar Fuels Laboratory, Nanyang Technological University, 50 Nanyang Avenue, Singapore, 639798 Singapore; 4 Institute of Chemical and Engineering Sciences, A*STAR, 1 Pesek Road, Singapore, 627833 Singapore; 5 Department of Materials Science and Engineering, University of California at Berkeley, Berkeley, CA 94720 USA; 6 Berkeley Educational Alliance for Research in Singapore Ltd., 1 CREATE Way, Singapore, 138602 Singapore; 7 Department of Chemical Engineering, University of Cambridge, Cambridge, CB2 3RA UK; 8 Energy Research Institute @ Nanyang Technological University, 50 Nanyang Avenue, Singapore, 639798 Singapore

## Abstract

Exploring robust catalysts for water oxidation in acidic electrolyte is challenging due to the limited material choice. Iridium (Ir) is the only active element with a high resistance to the acid corrosion during water electrolysis. However, Ir is rare, and its large-scale application could only be possible if the intrinsic activity of Ir could be greatly enhanced. Here, a pseudo-cubic SrCo_0.9_Ir_0.1_O_3-δ_ perovskite, containing corner-shared IrO6 octahedrons, is designed. The Ir in the SrCo_0.9_Ir_0.1_O_3-δ_ catalyst shows an extremely high intrinsic activity as reflected from its high turnover frequency, which is more than two orders of magnitude higher than that of IrO_2_. During the electrochemical cycling, a surface reconstruction, with Sr and Co leaching, over SrCo_0.9_Ir_0.1_O_3-δ_ occurs. Such reconstructed surface region, likely contains a high amount of structural domains with corner-shared and under-coordinated IrO_*x*_ octahedrons, is responsible for the observed high activity.

## Abstract (web summary)

While water splitting could provide a green means to store energy, there are few materials that can sustain high water oxidation half-reaction rates in acidic electrolytes. Here, authors design a perovskite oxide that generates high performance under-coordinated iridium sites during electrocatalysis.

## Introduction

The electricity from renewable energy sources, such as wind and solar power, has been showing a gradual proportional increase in global energy infrastructure^1^. However, due to the intermittent availability, the storage of the electric energy by these sources becomes pivotal. The conversion of the electricity into hydrogen fuel is expected to be one of the most efficient storage ways due to the high energy density of hydrogen fuel. Water electrolysis is a promising strategy to realize such energy conversion. The alkaline condition has been popular for industrial water electrolysis, while the acidic electrolyte condition is not. This is mainly due to the limited choices of robust anode catalysts in acidic condition during the oxygen evolution reaction (OER). In fact, the acidic condition offers a high proton concentration and thus enables much faster reaction kinetics of hydrogen production^2,3^. An ideal anode catalyst in acid should exhibit both high catalytic activity and high tolerance to the severe acidic corrosion at a high anodic potential. To date, the iridium oxides have been found high in stability in harsh acidic environment^4,5^. Due to the high cost and scarcity of Ir, great efforts have been made to develop novel Ir-based materials for water oxidation with high efficiency, e.g., a high activity normalized to the material surface area (intrinsic activity) and, more importantly, a high turnover frequency (TOF) for Ir^6–11^.

Recently, several Ir-based perovskites were reported as highly active catalysts for OER^12–15^. The perovskite is a type of oxide with a general formula of ABO_3_, where A represents alkaline-earth-metal or lanthanide and B represents active transition metals. Among these perovskite catalysts, a SrIrO_3_ perovskite, with reconstructed surface was found with the highest intrinsic activity (normalized to its surface area) for catalyzing OER in acidic environment to date^13^. Nevertheless, the synthesis of such SrIrO_3_ catalysts requires special tools or conditions, such as a pulse laser deposition (PLD) equipment^16^ or a high pressure (~5 GP) for solid state synthesis^17^, which could hinder the practical application of such catalyst. In addition, the key surface reconstruction step in this PLD-SrIrO_3_ is likely time-consuming because a ~30 h electrochemical activation process is required.

Here, we design a pseudo-cubic SrCo_0.9_Ir_0.1_O_3−δ_ material with an orthorhombic structure. In this design, the Ir and Co co-share the octahedral site and all octahedrons are corner shared. To form a comparison, we investigate a monoclinic SrIrO_3_ (m-SrIrO_3_) perovskite, which adopts a highly distorted 6H BaTiO_3_ structure with mixed corner-shared and face-shared IrO6 octahedrons^18,19^. We show that the intrinsic activity (TOF) of Ir from SrCo_0.9_Ir_0.1_O_3−δ_ is approximately two orders of magnitude higher than the m-SrIrO_3_ and more than one order of magnitude higher than the benchmark PLD-SrIrO_3_. The Sr and Co leach from SrCo_0.9_Ir_0.1_O_3−δ_ surface during electrochemical tests. As a result, a highly active surface IrO_*x*_ layer likely contains a high amount of corner-shared and under-coordinated IrO_*x*_ octahedrons without long-range ordering, and is responsible for the observed superior activity of SrCo_0.9_Ir_0.1_O_3−δ_.

## Results

### Structure characterization

**Fig. 1:**
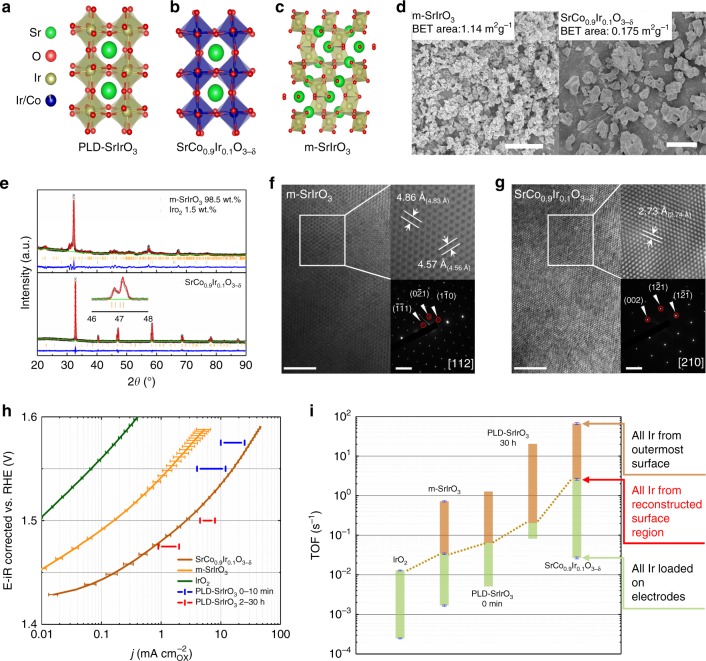
Crystal structure and OER activity of SrCo_0.9_Ir_0.1_O_3−δ_. Crystal structures of **a** PLD-SrIrO_3_^17^, **b** pseudo-cubic SrCo_0.9_Ir_0.1_O_3−δ_, and **c** m-SrIrO_3_. **d** scanning electron microscopy (SEM) images of as-prepared m-SrIrO_3_ (scale bar, 5 µm) and SrCo_0.9_Ir_0.1_O_3−δ_ (scale bar, 20 µm)_._ **e** Rietveld refinement of X-ray diffraction (XRD) patterns of as-prepared m-SrIrO_3_ and SrCo_0.9_Ir_0.1_O_3−δ_; minor IrO_2_ phase is found in the as-prepared m-SrIrO_3_ and a single SrCo_0.9_Ir_0.1_O_3−δ_ phase is obtained; the reliability factors for m-SrIrO_3_ are *R*_wp_ = 8.41%, *R*_p_ = 6.47%, *x*^2^ = 3.398; the reliability factors for the SrCo_0.9_Ir_0.1_O_3−δ_ are *R*_wp_ = 2.52%, *R*_p_ = 1.95%, *x*^2^ = 1.521. During the refinement, the structure parameters (space group: C *2/c*) of SrIrO_3_ reported by Qasim is used and only the lattice parameters of m-SrIrO_3_ were refined^19^. The refined lattice parameters for m-SrIrO_3_ are *a* = 5.5865(8), *b* = 9.648(1), *c* = 14.153(1), alpha = 90°, beta = 93.048(9)°, and gamma = 90°. The detailed structure parameters for SrCo_0.9_Ir_0.1_O_3−δ_ are presented in Supplementary Table 1. A refined occupancy ratio for Ir in SrCo_0.9_Ir_0.1_O_3−δ_ is 0.093, which confirms the Ir has been successfully doped in the Co site. **f**, **g** High-resolution transmission electron microscopy (HRTEM) images taken from m-SrIrO_3_ and SrCo_0.9_Ir_0.1_O_3−δ_ (scale bar, 5 nm). The corresponding inverse fast Fourier transformed (FFT) images are taken from white square box regions (scale bar, 5 1/nm). The measured interplanar distances from inverse FFT images correspond well with the XRD refinement results, which are in brackets. The selected area electron diffraction (SAED) patterns from corresponding zone axes are presented and indexed based on the data from XRD refinement. **h** Specific OER activities of IrO_2_, m-SrIrO_3_, and SrCo_0.9_Ir_0.1_O_3−δ_ in 0.1 M HClO_4_. The specific OER activities of PLD-SrIrO_3_ in 0.5 M H_2_SO_4_ are from the literature^13^. For the activity of PLD-SrIrO_3_ samples, the activity evolution within 0–10 min and 2–30 h are shown as bars. **i** Calculated range for turnover frequency (TOFs) of Ir from different samples at an overpotential of 270 mV. The TOFs are calculated by assuming Ir at outermost surfaces (upper limit), Ir from reconstructed surface regions (the intermediate value) and all Ir from the bulk (lower limit) are involved in catalyzing water oxidation. The error bars correspond to the s.d

Figure 1a–c shows the crystal structures of PLD-SrIrO_3_, pseudo-cubic SrCo_0.9_Ir_0.1_O_3−δ_, and m-SrIrO_3_. In PLD-SrIrO_3_ and pseudo-cubic SrCo_0.9_Ir_0.1_O_3−δ_, all IrO6 octahedrons are corner-shared. Nevertheless, in m-SrIrO_3_, corner-shared and face-shared IrO6 octahedrons are alternatively arranged. Both m-SrIrO_3_ and SrCo_0.9_Ir_0.1_O_3−δ_ samples were synthesized with a classical solid state method, which is easily accessible for large scale preparation. Figure 1d includes the scanning electron microscope (SEM) images from m-SrIrO_3_ and SrCo_0.9_Ir_0.1_O_3−δ_ samples. The corresponding Brunauer–Emmett–Teller (BET) surface area for these two samples are also presented. The SrCo_0.9_Ir_0.1_O_3−δ_ has a lower BET surface area due to its relative large particles. A m-SrIrO_3_ with a space group (SG) of C *2/c* and a SrCo_0.9_Ir_0.1_O_3−δ_ with SG of P *nma* are confirmed based on their X-ray diffraction (XRD) patterns (the refined results are shown in Fig. 1e and Supplementary Table 1). Figure 1f, g presents HRTEM images taken from m-SrIrO_3_ and SrCo_0.9_Ir_0.1_O_3−δ_. The corresponding inverse fast Fourier transformed (FFT) images are taken from white square box regions. The measured interplanar distances from inverse FFT images correspond well with the results from XRD refinement, which are in brackets. Selected area electron diffraction (SAED) patterns are also presented. All patterns can be well indexed based on the XRD refinement results, indicating the formation of m-SrIrO_3_ and SrCo_0.9_Ir_0.1_O_3−δ_ with high purity.

### Activity evaluation

The activities of SrCo_0.9_Ir_0.1_O_3−δ_, m-SrIrO_3_ and commercial IrO_2_ for the OER were then evaluated in a perchloric acid solution (0.1 M, pH = 1.08). The measured specific activity (mA cm^−2^ normalized to BET surface area), representing the intrinsic activity of a catalyst^20^, is shown in Fig. 1h. The activity for the PLD-SrIrO_3_ prepared with PLD method from literature is also presented for comparison^13^. The calculated specific activity of IrO_2_ is similar to the previously reported values^7^. A higher specific activity, approximately one order of magnitude higher than that of IrO_2_, is obtained from the m-SrIrO_3_. For instance, at a potential of 1.5 V (vs. RHE), a specific current density of ~1 × 10^−2^ mA cm^−2^ and ~0.2 mA cm^−2^ is recorded for IrO_2_ and m-SrIrO_3_, respectively. However, such activity is much inferior to that of the PLD-SrIrO_3_. Moreover, different from the reported PLD-SrIrO_3_ catalyst, the m-SrIrO_3_ did not show a gradually increased activity during the successive test (Supplementary Figure 1). As a result, the specific activity of the m-SrIrO_3_ is more than one order of magnitude lower than that from the PLD-SrIrO_3_ catalyst (stabilized activity at 2 h). This activity difference indicates that the pseudo-cubic structure is a key factor to determine the intrinsic activity of SrIrO_3_. On the other hand, regardless of the low Ir atom ratio in the lattice, the specific activity of SrCo_0.9_Ir_0.1_O_3−δ_ is more than one order of magnitude higher than that from m-SrIrO_3_ and comparable with the reported initial activity of PLD-SrIrO_3_. Interestingly, such specific activity is just 2–4 times lower than the final specific activity of PLD-SrIrO_3_, which hints the intrinsic activity of Ir over the surface of SrCo_0.9_Ir_0.1_O_3−δ_ is higher than that of Ir over the surface of PLD-SrIrO_3_. We caution that the Co in SrCo_0.9_Ir_0.1_O_3−δ_ does not contribute to the observed superior activity of SrCo_0.9_Ir_0.1_O_3−δ_ for its high solubility in the acidic electrolyte (Supplementary Figure 2). Additionally, as discussed in the following sections, a rapid dissolution of Sr and Co is expected in acidic electrolytes. The stable activity of SrCo_0.9_Ir_0.1_O_3−δ_ during cycling test, shown in Supplementary Figure 2, also confirms that the measured OER current contributed by Sr and Co leaching is negligible. In addition, an estimated maximum current due to Sr and Co leaching in initial 5 cycles is more than two orders of magnitude lower than the measured OER current from SrCo_0.9_Ir_0.1_O_3−δ_ (see the estimation of the current due to Sr and Co leaching in the Methods). Figure 1i presents the TOF of Ir from IrO_2_, m-SrIrO_3_, and SrCo_0.9_Ir_0.1_O_3−δ._ Three cases, including Ir at outermost surfaces (upper limit), of Ir from reconstructed surface regions (the surface reconstruction over catalysts will be discussed later) and all Ir from the bulk (lower limit), were considered for TOF calculations. Calculation details are presented in Methods. Among them, the TOF of Ir by considering the effect of surface reconstruction should be the most reliable for comparing the intrinsic activity of Ir from different materials. From Fig. 1i, at an overpotential of 270 mV (1.5 V vs. RHE), the Ir in SrCo_0.9_Ir_0.1_O_3−δ_ is found with a high TOF of 2.56 ± 0.15 s^−1^, which is more than 10 times and ~75 times higher than PLD-SrIrO_3_ (~0.2 s^−1^) and m-SrIrO_3_ (0.034 ± 0.001 s^−1^), respectively. It confirmed that the observed superior activity of SrCo_0.9_Ir_0.1_O_3−δ_ is attributed to the formation of highly active surface Ir species.

### Surface reconstruction

**Fig. 2:**
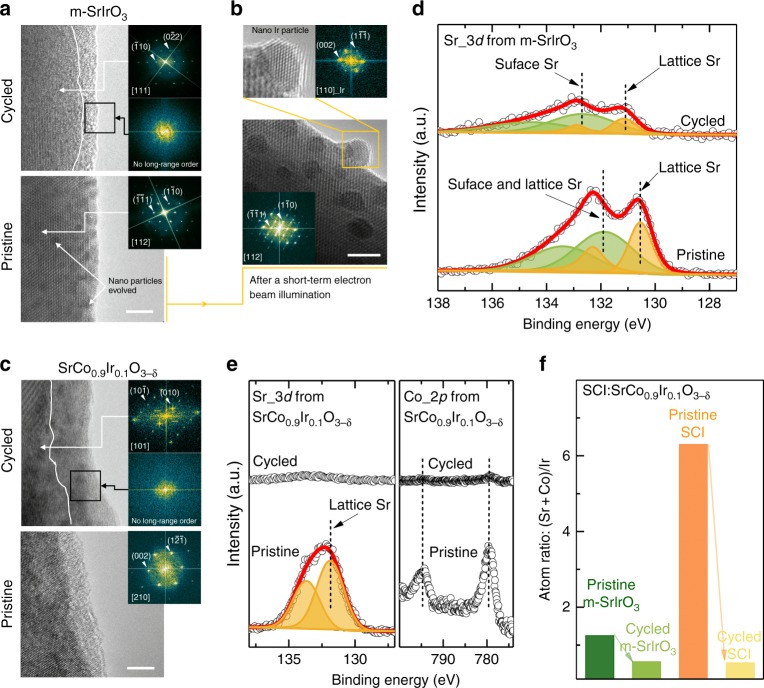
Analysis of surface structure reconstruction. **a** High-resolution transmission electron microscopy (HRTEM) images of pristine and cycled m-SrIrO_3_ (by 5 cycles, scale bar, 5 nm). **b** The HRTEM image of the exsolution of nanoparticles with increased size from m-SrIrO_3_ after a short-term electron beam illumination. The fast Fourier transformed (FFT) pattern from the nanoparticle can be indexed to a F *m*$\overline 3$*m* space group of Ir metal. The exsolution of nano Ir particles has no apparent effect on the crystallinity of surface region. **c** HRTEM images of pristine and cycled SrCo_0.9_Ir_0.1_O_3−δ_ (by 5 cycles, scale bar, 5 nm). The white curves in **a** and **c** indicate the interfaces between the crystallized region and the reconstructed region. The FFT patterns from the bulk of corresponding HRTEM images can be well indexed based on space groups of C *2/c* for m-SrIrO_3_ and P *nma* for SrCo_0.9_Ir_0.1_O_3−δ_. As reflected in the FFT patterns from the surfaces of cycled m-SrIrO_3_ and cycled SrCo_0.9_Ir_0.1_O_3−δ_, no long-range order exists in the surface regions. **d** Sr_3*d* XPS for m-SrIrO_3_ before and after 5 cyclic voltammetry (CV) cycles. **e** Sr_3*d* and Co_2*p* XPS for SrCo_0.9_Ir_0.1_O_3−δ_ before and after the electrochemical tests. **f** The surface (Sr + Co):Ir ratio in m-SrIrO_3_ and SrCo_0.9_Ir_0.1_O_3−δ_ before and after the electrochemical cycling

For IrO_2_, it has been reported with a high resistance to acidic corrosion^21^. However, it is unlikely that m-SrIrO_3_ and SrCo_0.9_Ir_0.1_O_3−δ_ can keep their original structure in strong acidic condition due to the thermodynamic instability of Sr and Co. The surface structure evolution of m-SrIrO_3_ and SrCo_0.9_Ir_0.1_O_3−δ_ is then evaluated by HRTEM. Moreover, since the performance of both SrCo_0.9_Ir_0.1_O_3−δ_ and m-SrIrO_3_ become stable in a few cycles (within 5 cycles), the surface structures of these two materials after 5 cycles were also investigated. The HRTEM images from pristine and cycled m-SrIrO_3_ are presented in Fig. 2a. No apparent structural reconstruction can be observed from the surface of pristine m-SrIrO_3_, while a surface reconstruction from cycled m-SrIrO_3_ is observed with a depth of approximately 5 nm. By comparing the FFT images from bulk and surface, such reconstructed surface is likely amorphous with no long-range order. We caution that nanoparticles (~2 nm) emerge from the bulk of the m-SrIrO_3_ after a short-term electron beam illumination, and such nanoparticles were determined to be Ir (Fig. 2b). Similar behaviors have also been observed in a few other Ir-based oxides^22,23^. In spite of the formation of Ir nanoparticles, the crystallinity of the surfaces of m-SrIrO_3_ was not affected during the period of TEM analysis. Figure 2c presents the HRTEM images from pristine and cycled SrCo_0.9_Ir_0.1_O_3−δ_. The surface region from pristine SrCo_0.9_Ir_0.1_O_3−δ_ with a depth of 1–3 nm is amorphous. Such slight surface amorphization in pristine SrCo_0.9_Ir_0.1_O_3−δ_ likely occurs during the TEM sample preparation process, in which the sample is ultrasonically dispersed in ethanol solution. After electrochemical cycling, an apparent surface structure reconstruction is observed for SrCo_0.9_Ir_0.1_O_3−δ_ with a depth of approximately 10 nm. Similar to the case in m-SrIrO_3_, such reconstructed surface of SrCo_0.9_Ir_0.1_O_3−δ_ is also likely amorphous with no long range order. The FFT image from bulk of SrCo_0.9_Ir_0.1_O_3−δ_ can be well indexed, indicating the bulk maintains its initial crystal structure. In combination with the observed reconstructed surface layers, the in situ reconstructed surface layers are responsible for the observed activities from the m-SrIrO_3_ and the SrCo_0.9_Ir_0.1_O_3−δ_.

The X-ray photoelectron spectroscopy (XPS) was then performed to study the surface chemical states in m-SrIrO_3_ and SrCo_0.9_Ir_0.1_O_3−δ_ before and after 5 cyclic voltammetry (CV) cycles. The XPS of Sr_3*d* from the m-SrIrO_3_ are shown in Fig. 2d. The fitting parameters are listed in Supplementary Table 2. To compare the relative composition change, all Sr_3*d* spectra are normalized based on corresponding Ir contents. In Fig. 2d, an apparent decrease in Sr_3*d* signal is observed from cycled m-SrIrO_3_, indicating a Sr leaching during the test. Moreover, although the Sr_3*d* signal from pristine m-SrIrO_3_ and cycled m-SrIrO_3_ can both be fit with two doublets, their profile is totally different. Specifically, both doublets in the pristine m-SrIrO_3_ should be mainly related to the Sr from m-SrIrO_3_ lattice as two low binding energy of 130.55 and 131.81 eV for the 3*d*5/2 peak are obtained^24^. In addition, a relative larger full width at half maximum (FWHM) for the second doublet (marked in green) indicates it also includes a small amount of surface Sr components, such as SrO, SrCO_3_, and Sr(OH)_2_, with higher binding energies (above 133 eV)^25^. In the cycled m-SrIrO_3_, the first doublet (marked in yellow) with a low binding energy of 131.15 eV should still be related to lattice Sr. More surface Sr components seem formed because the second doublet shifts to a higher binding energy of 132.62 eV for the 3*d*5/2 peak. In combination with the HRTEM results, the increased amount of surface Sr components is likely related to the leached bulk Sr, which re-deposits on the electrode surface. Figure 2e shows the XPS of Sr_3*d* and Co_2*p* from pristine and cycled SrCo_0.9_Ir_0.1_O_3−δ_ samples. The fitting parameters for Sr_3*d* spectra are listed in Supplementary Table 3. In the spectrum of Sr_3*d* from pristine SrCo_0.9_Ir_0.1_O_3−δ_ sample, only one doublet at 131.83 eV for the 3*d*5/2 peak is observed, which should be mainly related to lattice Sr. Importantly, a severe Sr leaching is observed from the cycled SrCo_0.9_Ir_0.1_O_3−δ_ sample as the Sr_3*d* signal is almost invisible just after 5 CV cycles (time of duration: ~10 min). Similarly, a fast cobalt leaching is observed from the surface of cycled SrCo_0.9_Ir_0.1_O_3−δ_ because the XPS signal of Co_2*p* from cycled SrCo_0.9_Ir_0.1_O_3−δ_ sample is almost invisible. As summarized in Fig. 2f, after 5 cycles, approximately 50% Sr dissolved from the surface of m-SrIrO_3_. In the reported PLD-SrIrO_3_ catalyst, approximately 75% Sr is expected to leach from its surface after 30 min of electrochemical OER testing^13^. Although the leaching degree of Sr from the surface of m-SrIrO_3_ is slightly lower than that in PLD-SrIrO_3,_ considering the much inferior activity of m-SrIrO_3_, the initial pseudo-cubic structure with corner-shared IrO6 octahedron is critical for a highly active Ir site after the cation leaching. This corresponds well with the previous calculation results^13^, which found the reconstructed surface with a structure similar to IrO_3_ or anatase IrO_2_ is active to catalyze OER. Different from m- and PLD-SrIrO_3_, the surface region of SrCo_0.9_Ir_0.1_O_3−δ_ loses approximately 92% Sr + Co after 5 CV cycles. This Sr and Co leaching in acid from SrCo_0.9_Ir_0.1_O_3−δ_ likely causes the obvious surface reconstruction on SrCo_0.9_Ir_0.1_O_3−δ_. More importantly, we can confirm the formed amorphous surface Ir-related species after Sr and Co leaching are responsible for the measured activity of m-SrIrO_3_ and SrCo_0.9_Ir_0.1_O_3−δ_.

### Understanding Ir from reconstructed surfaces

**Fig. 3:**
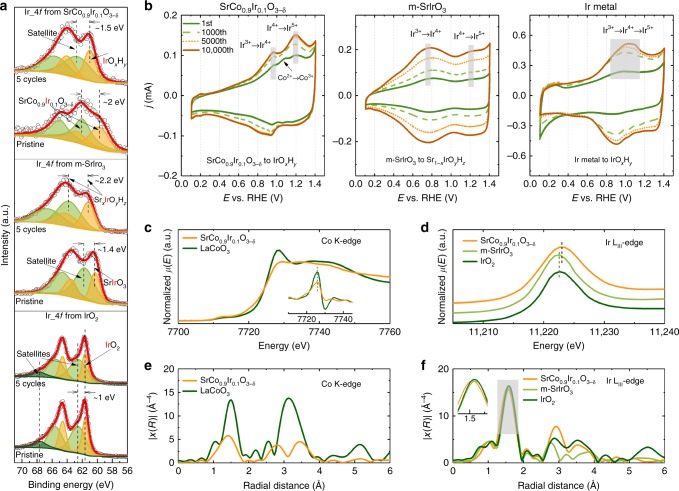
Evaluation of the formed IrO_*x*_ phase(s) and SrCo_0.9_Ir_0.1_O_3−δ_ bulk chemical states. **a** Ir_4*f* X-ray photoelectron spectroscopy (XPS) for IrO_2_, m-SrIrO_3_, and SrCo_0.9_Ir_0.1_O_3−δ_ before and after the electrochemical tests. **b** The cyclic voltammetry (CV) curves recorded at the 1st, 1000th, 5000th, and 10,000th cycles for SrCo_0.9_Ir_0.1_O_3−δ_, m-SrIrO_3_, and Ir metal in a 0.1 M HClO_4_ electrolyte. The cycles presented are collected at a scan rate of 100 mV s^−1^. For the intermediate cycles, a scan rate of 1 V s^−1^ is applied. **c** X-ray absorption near edge structure (XANES) and 1st derivative XANES spectra (inset) of LaCoO_3_ and SrCo_0.9_Ir_0.1_O_3−δ_ measured at Co K-edge. **d** XANES spectra of IrO_2_, m-SrIrO_3_, and SrCo_0.9_Ir_0.1_O_3−δ_ measured at Ir L_III_-edge. **e** k^3^-weighted Co K-edge Extended X-Ray Absorption Fine Structure (EXAFS) spectra of LaCoO_3_ and SrCo_0.9_Ir_0.1_O_3−δ_. **f** k^3^-weighted Ir L_III_-edge EXAFS spectra of IrO_2_, m-SrIrO_3_, and SrCo_0.9_Ir_0.1_O_3−δ_

Ir from IrO_2_, m-SrIrO_3_, and SrCo_0.9_Ir_0.1_O_3−δ_ were investigated by XPS to gain more information about the Ir in reconstructed surfaces and the Ir_4*f* spectra from the three samples before and after electrochemical tests are given in Fig. 3a. The Ir_4*f* spectra from the IrO_2_ are studied first. Distinctive asymmetric tail can be observed from the peaks in the two spectra and is reported related to the screening response of 5*d* conduction electrons^26^. Based on the previous studies, both Ir_4*f* spectra can be fitted with two doublets and an additional single satellite peak. The fitting results are listed in Supplementary Table 4. The Ir_4*f*7/2 peak with a binding energy of 61.61 and 61.63 eV is found from the first doublet (marked in yellow) in the pristine IrO_2_ and the cycled IrO_2_, respectively. The observed binding energies are within the reported ranges for Ir_4*f*7/2 peak in IrO_2_ (61–62 eV). The second wide doublet (marked in green), with an energy shift of approximately 1 eV as compared with the first doublet, should be related to the shake-up satellites of the first doublet^27^. The spin–orbit splitting in both doublets is 2.98–2.99 eV, which is also close to the reported values^27–29^. The additional single satellite at 67.6 eV for pristine IrO_2_ or at 67.76 eV for the cycled IrO_2_ is related to the localized non-bonding states^27,28^. From the fitting results, the Ir 4*f* spectra from the IrO_2_ is almost unchanged after the electrochemical cycling, confirming a highly stable surface structure of IrO_2_ during OER in acidic environment.

The Ir_4*f* spectra from the m-SrIrO_3_ were also fit to explore the change of surface Ir after electrochemical cycling. The fitting parameters are listed in Supplementary Table 2. Both spectra can be fit with two doublets. In the pristine m-SrIrO_3_, the Ir_4*f*7/2 peak from the first doublet located at a low binding energy of 60.33 eV, which is generally considered as the signal of Ir metal^30^. However, Ir metal is unlikely to exist in the sample as a long-term high temperature calcination is required in the solid state method for sample preparation. Considering the Ir in the m-SrIrO_3_ is 4+ for charge neutrality, it is rational to define this first doublet comes from Ir^4+^. Moreover, in the previous studies, the Ir_4*f*7/2 peak of Ir^4+^ from PLD-SrIrO_3_ is found with a binding energy located in between 61.8 and 62.6 eV^13^. The much lower binding energy observed in the m-SrIrO_3_ indicates the XPS signal of Ir is strongly related to its local structural environment. The second doublet has a binding energy ~1.4 eV higher than that of the first doublet. This energy shift between two doublets is similar to the cases observed in rutile IrO_2_ and PLD-SrIrO_3_, indicating the second doublet should be shake-up satellites. After electrochemical cycling, apparent shifts of the two doublets in the Ir_4*f* spectra to higher binding energy are observed. The Ir_4*f*7/2 in the first doublet is at 61.2 eV, which is approximately 0.9 eV higher than that in pristine m-SrIrO_3_. This moderate energy shift is different from the case observed in PLD-SrIrO_3_, in which the Ir_4*f*7/2 in the first doublet is almost unchanged irrespective of the Sr leaching during electrochemical testing^13^. The Sr leaching in m-SrIrO_3_ probably influences the chemical state of Ir and thus certain Sr_*x*_IrO_*y*_H_*z*_ (*x* < 1) phase(s) may form, considering a high amount of Sr remains in the surface. The Ir_4*f*7/2 peak from the second doublet has a high binding energy of 63.35 eV, which, however, cannot be solely treated as the shake-up satellites because an energy shift of approximately 2.2 eV, relative to the first doublet, is observed. Such energy shift is much larger than those (1–1.6 eV) observed in rutile IrO_2_, PLD-SrIrO_3_ as well as the pristine m-SrIrO_3_. The unusual energy shift of this second doublet hints the formed surface is very complex and multiple phases can exist.

The profile of Ir_4*f* spectra from the pristine SrCo_0.9_Ir_0.1_O_3−δ_ is different from those observed in rutile IrO_2_ or SrIrO_3_ phases. The fitting parameters are listed in Supplementary Table 3. The fitting results revealed an Ir_4*f*7/2 peak at approximately 59.7 eV for the first doublet. This Ir_4*f*7/2 peak with a large FWHM likely represents the mixed Ir^4/5+^ in SrCo_0.9_Ir_0.1_O_3−δ_ since a low binding energy is also observed in the pristine m-SrIrO_3_ phase. The second doublet with a binding energy ~2 eV higher than the first doublet cannot be solely treated as the shake-up satellite. Certain Ir specie(s) from SrCo_0.9_Ir_0.1_O_3−δ_ surface can exist, and its identification is beyond the scope of this study. After electrochemical cycling, the Ir_4*f* spectra from SrCo_0.9_Ir_0.1_O_3−δ_ can be well fit with two doublets. A binding energy of 61.0 eV is observed for the Ir_4*f*7/2 peak in the first doublet. Such peak position is more than 1 eV higher than that in pristine SrCo_0.9_Ir_0.1_O_3−δ_, but ~0.6 eV lower than that in rutile IrO_2_. The second doublet in cycled SrCo_0.9_Ir_0.1_O_3−δ_ shifts ~1.5 eV to higher binding energy, which then can be related to the shake-up satellites. As a result, certain IrO_*x*_H_*y*_ phase may form over the SrCo_0.9_Ir_0.1_O_3−δ_ surface as Sr and Co have leached out. Previously, some amorphous IrO_*x*_ phases have also been prepared for a higher activity than crystallized rutile IrO_2_^22,31,32^. However, the Ir_4*f* spectra from amorphous IrO_*x*_ and crystalline rutile IrO_2_ always have similar peak positions and profiles^27,31–33^. Moreover, the influence of Sr on Ir_4*f* in XPS can be excluded since there is almost no Sr in the surface region after five cycles (Fig. 2e). In fact, the signals of Sr and Co in XPS from the surface region of SrCo_0.9_Ir_0.1_O_3−δ_ disappeared completely after 10k cycles (Supplementary Figure 3a & b). That indicates only Ir remaining in the surface region. Therefore, the possibility that the residual Sr caused the difference (Fig. 3a and Supplementary Figure 3c) in Ir_4*f* between cycled SrCo_0.9_Ir_0.1_O_3−δ_ and hydrous IrO_*x*_ can be excluded. As a result, the formed IrO_*x*_H_*y*_ phase in SrCo_0.9_Ir_0.1_O_3−δ_ is different from previous amorphous IrO_*x*_ phases.

For a better understanding of the formation and evolution of the surface in m-SrIrO_3_ and SrCo_0.9_Ir_0.1_O_3−δ_, we further studied the CV cycles measured from SrCo_0.9_Ir_0.1_O_3−δ_ and m-SrIrO_3_. Since Ir hydrous oxide can be formed on metal Ir through electrochemical cycling, the CVs of Ir metal was also recorded for comparison^34^. As shown in Fig. 3b, different redox behaviors are observed for Ir from these three catalysts. For SrCo_0.9_Ir_0.1_O_3−δ_, three oxidation peaks, representing Ir^3+/4+^ (~1 V), Co^2+/3+^ (~1 V), and Ir^4+/5+^ (~1.2 V), can be observed at the first cycle^35,36^. In the following cycles, the Co peak disappeared and only the two peaks belonging to Ir become remarkable, which is probably due to the dissolution of Co as well as the formation of IrO_*x*_H_*y*_ species in the surface region. The intensities of the two Ir-related oxidation peaks simultaneously increased, which should be related to the increased degree of surface reconstruction. Interestingly, the surface reconstruction process likely stopped after 5000 cycles since the 5,000th cycle almost overlapped with the 10,000th cycle. The peak at ~1.2 V indicates the oxidation of Ir^4+^ to Ir^5+^ is greatly facilitated over the SrCo_0.9_Ir_0.1_O_3−δ_ surface. For m-SrIrO_3_, two oxidation peaks, representing Ir^3+/4+^ (~0.8 V) and Ir^4+/5+^ (~1.2 V), can be observed. However, unlike SrCo_0.9_Ir_0.1_O_3−δ_, the redox of Ir^3+^/Ir^4+^ is dominant on m-SrIrO_3._ As for Ir metal, one distinctive oxidation peak at ~1 V is observed after ~1000 cycles, indicating the formation of surface Ir hydrous oxide. Such broad oxidation peak likely due to successive oxidation of Ir^3+^ to Ir^5+^^35^. By comparing the CVs of these three catalysts, one can conclude that they give different Ir-based amorphous phase(s) by cycling. The formation of different Ir-related phase(s) over these catalysts is also indicated by corresponding XPS results (Supplementary Figure 3 and Supplementary Table 5). More importantly, the profiles of CVs (the 5000th/10,000th cycle) from cycled samples resemble those of 1st cycle, strongly suggest that the formed Ir-based amorphous phase(s) are strongly influenced by the initial lattice.

### Possible active phase after surface reconstruction

Till now, several facts have been put forward to explain the high activities measured from Ir-based complex oxides with perovskite or perovskite-related structures^12–15^. For example, derived from Sr-leached SrIrO_3_, it is revealed certain IrO_*x*_, with structure resemble cubic perovskite, is highly active towards OER^13^. A similar conclusion was obtained from Ir-based double perovskites, that is Ir is more active in 3D network of corner-shared octahedrons^12^. Although the surfaces of these double perovskites may also experience fast surface rearrangement, the initial cubic perovskite structure with corner-shared octahedrons seems to be a key factor that influence or even determine the activity of formed surface active phase(s). This may also explain the inferior activity of m-SrIrO_3_ in our study. In fact, for an amorphous IrO_*x*_, the strong correlation between activity and local structure (corner-shared vs. edge-shared IrO6 octahedrons) has also been highlighted by Willinger et al.^23^. Besides, it is reported that certain lattice oxygen atom can be activated in a La_2_LiIrO_6_ perovskite. Such activated lattice oxygen atom can participate in the OER and is proposed critical for the observed high activity^15^. However, as surface reconstruction is observed in our catalysts, the activated lattice oxygen atom from perovskite is unlikely. We hypothesized that the high activity of IrO_*x*_H_*y*_ evolved from SrCo_0.9_Ir_0.1_O_3−δ_ due to the strong correlation between the IrO_*x*_H_*y*_ and the initial/bulk lattice of SrCo_0.9_Ir_0.1_O_3−δ_.

In the developed SrCo_0.9_Ir_0.1_O_3−δ_, first of all, its pseudo-cubic structure with initially corner-shared octahedrons can induce the formation of a local structure optimized IrO_*x*_H_*y*_ phase, which still contains a high ratio of corner-shared IrO6 octahedrons. However, this is insufficient to explain the measured higher activity when compared with that from the reported PLD-SrIrO_3_^13^. As compared with SrIrO_3_ and other reported Ir-based perovskites, the SrCo_0.9_Ir_0.1_O_3−δ_ here is expected to possess a high amount of oxygen vacancies in the lattice since the SrCoO_3−δ_ matrix is highly oxygen deficient^37^. Accordingly, the oxygen vacancy formation energies for m-SrIrO_3_, PLD-SrIrO_3_, and SrCo_0.9_Ir_0.1_O_3_ were calculated by density functional theory (DFT). Moreover, the O *p*-band centers of different materials were also calculated. This is because a perovskite with an O *p*-band center close to Fermi level was found easier to release oxygen, i.e., the formation of oxygen vacancies^38^ (computation details and discussions are shown in Supplementary Figure 4&5 and Supplementary Table 6). As compared with m-SrIrO_3_ and PLD-SrIrO_3_, the DFT calculations indicate that the SrCo_0.9_Ir_0.1_O_3−δ_ could contain a higher amount of oxygen vacancies.

To further investigate the oxygen vacancies in SrCo_0.9_Ir_0.1_O_3−δ_, the X-ray adsorption spectroscopy (XAS) was performed to check the valence state and coordination environment of Co and Ir. As shown in Fig. 3c, the average valence state of Co in the SrCo_0.9_Ir_0.1_O_3−δ_ is approximately 3+ by comparing with the Co K-edge from the standard LaCoO_3_, in which the Co is strictly trivalent state^39,40^. Considering the Ir-L_III_ edge positions from m-SrIrO_3_ and IrO_2_ are similar to each other (Fig. 3d), the oxidation state of Ir in m-SrIrO_3_ is approximately 4+, indicating almost no oxygen vacancy in m-SrIrO_3_. The valence state of Ir in SrCo_0.9_Ir_0.1_O_3−δ_ is found slightly higher than 4+ as the Ir-L_III_ edge shifts to higher energy as compared with IrO_2_. For charge neutrality, the δ (oxygen nonstoichiometry) in SrCo_0.9_Ir_0.1_O_3−δ_ can reach a minimum value of 0.4 if assuming all Ir is pentavalent state. Therefore, a high amount of oxygen vacancies should exist in SrCo_0.9_Ir_0.1_O_3_, and thus both Co and Ir in SrCo_0.9_Ir_0.1_O_3−δ_ are highly under-coordinated. Accordingly, the Fourier transformed (FT) EXAFS spectra in R-space for Co and Ir in SrCo_0.9_Ir_0.1_O_3−δ_ are compared with the spectra from standard LaCoO_3_, m-SrIrO_3_, and IrO_2_, in which Co and Ir are fully or almost fully coordinated. In FT EXAFS spectra, the peaks are related to the coordination shells of Ir and Co. Specifically, the first peak represents the first Ir/Co–O shell caused by the interference between the electronic back scatterings from Ir/Co to neighbor O. Figure 3e shows the FT EXAFS spectra of the Co K edge from SrCo_0.9_Ir_0.1_O_3−δ_. The first peak at a reduced distance of ~1.5 Å represents the first Co–O coordination shell in SrCo_0.9_Ir_0.1_O_3−δ_ and it shows a much lower intensity as compared with the first peak from LaCoO_3_, indicating the highly under-coordinated Co. Additional evidence of such high oxygen-deficiency is obtained from the fitting of the first peaks, where the coordination number of Co in SrCo_0.9_Ir_0.1_O_3−δ_ is approximately 4.1 (Supplementary Figure 6 and Supplementary Table 7). The comparison of FT EXAFS spectra at the Ir L_III_-edge from IrO_2_, m-SrIrO_3_, and SrCo_0.9_Ir_0.1_O_3−δ_ is shown in Fig. 3f. The first peak (at ~1.5 Å) from IrO_2_ and m-SrIrO_3_ overlaps to each other, while a shift to lower distance (shown in the inset of Fig. 3f) is found for SrCo_0.9_Ir_0.1_O_3−δ_, implying a shorter average Ir–O bond length in SrCo_0.9_Ir_0.1_O_3−δ_. The fitting of first Ir–O coordination shells also confirms this reduced bond length. (Supplementary Figure 7 and Supplementary Table 8). As expected, a first shell fitting indicates Ir in SrCo_0.9_Ir_0.1_O_3−δ_ is also highly under-coordinated with a coordination number of ~4.9. Instead, the Ir in m-SrIrO_3_ is fully coordinated, which is consistent with the DFT result.

**Fig. 4:**
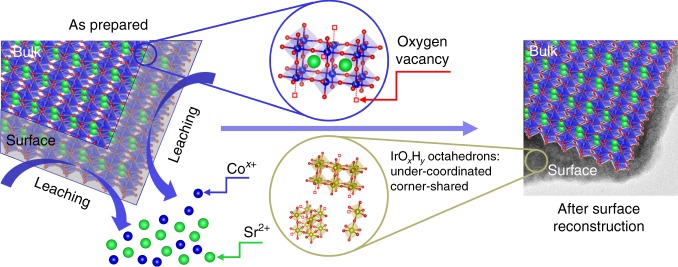
Surface reconstruction. A schematic that illustrates the surface reconstruction over the SrCo_0.9_Ir_0.1_O_3−δ_ surface

Based on the results shown in Figs. 2 and 3, a schematic in Fig. 4 is proposed to illustrate the possible surface reconstruction over the SrCo_0.9_Ir_0.1_O_3−δ_ surface. In initial SrCo_0.9_Ir_0.1_O_3−δ_, the IrO6 octahedrons are corner-shared with surrounding (Co/Ir)O6 octahedrons. Due to the presence of oxygen vacancies, the Ir in the lattice is under-coordinated. During electrochemical cycling, fast leaching of Sr and Co leaves an Ir rich surface. The initial pseudo-cubic structure will collapse on the surface and the formed IrO_*x*_H_*y*_ phase(s) is amorphous without long-range ordering. The formed IrO_*x*_H_*y*_ phase(s) may contain a high amount of structural domains with corner-shared IrO6 octahedrons. Moreover, the Ir in the amorphous phase could be more under-coordinated as compared with those derived from SrIrO_3_ after Sr leaching. This deduction also explains why the activity of Ir from SrCo_0.9_Ir_0.1_O_3−δ_ is higher than that of Ir from electrochemically cycled SrIrO_3_.

### Assessing the stability of SrCo_0.9_Ir_0.1_O_3−δ_

**Fig. 5:**
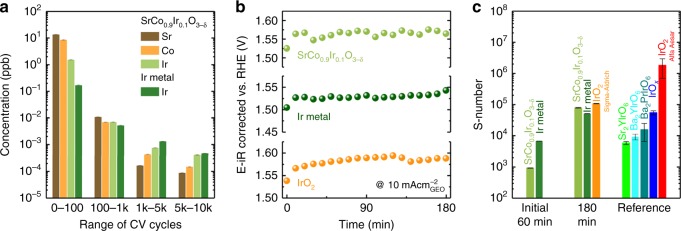
Stability of SrCo_0.9_Ir_0.1_O_3−δ_. **a** The averaged concentration of dissolved cations per cycle at different stages. The scan rate is 1 V s^−1^. For the SrCo_0.9_Ir_0.1_O_3−δ_ electrode, the potential range is 0.1–1.4 V (vs. RHE). As to the Ir metal electrode, to improve the Ir dissolution, the potential range for the initial 1k cycles and the rest 9k cycles is 0.1–1.6 V (vs. RHE) and 0.1–1.8 V (vs. RHE), respectively. Moreover, a higher overpotential (1.8 V vs. 1.6 V) applied in the rest 9k cycles for Ir metal can facilitate the Ir dissolution^41^. However, we can see that after 1k cycles, even the upper limit is increased to 1.8 V for Ir metal, there is no significant increase of Ir dissolution within the following 9k cycles. Instead, a lower Ir dissolution is observed, indicating a high instability of Ir at the early stage. **b** Potential profiles of different electrodes. The chronopotentiometry is performed in 0.1 M HClO_4_ at 10 mA cm^−2^, which is normalized to the geometric area of electrodes (GEO). Please note that it does not reflect the intrinsic activity of these materials. **c** The calculated S-numbers of SrCo_0.9_Ir_0.1_O_3−δ_, Ir metal, and IrO_2_. The S-numbers of some Ir-based perovskites reported by Geiger et al. are also presented^41^

Most recently, Geiger et al. proposed a stability number (S-number), considering the dissolution of active sites, is a proper metric to evaluate the stability of catalysts^41^. Thus, in this study, we also calculate the S-number of Ir in SrCo_0.9_Ir_0.1_O_3−δ_. The dissolution of cations, including Sr, Co, and Ir, from SrCo_0.9_Ir_0.1_O_3−δ_ during electrochemical tests are studied. The amount of dissolved cations during the cycling are shown in Fig. 5a. The corresponding overall dissolved Sr, Co, and Ir from SrCo_0.9_Ir_0.1_O_3−δ_ during the cycling are shown in Supplementary Figure 8. The dissolution of Ir from Ir metal is also measured for comparison. At the early stage (0–100 cycles), fast Sr, Co, and Ir dissolution is observed from SrCo_0.9_Ir_0.1_O_3−δ_. The simultaneous dissolution of Sr and Co likely induces such high dissolution rate of Ir. In the following cycles, the dissolution rate of Ir from SrCo_0.9_Ir_0.1_O_3−δ_ is found slowed down. Interestingly, the apparently reduced dissolution rates of Sr and Co suggests that the dissolution of Sr and Co from SrCo_0.9_Ir_0.1_O_3−δ_ is kinetically hindered by the formed Ir-rich surface layer. Similarly, The Ir metal also showed a gradually decreased rate of Ir dissolution. That is the formation of active Ir hydrous oxide also causes a fast Ir dissolution at the early stage. Figure 5b shows the potential profiles of as-synthesized/purchased SrCo_0.9_Ir_0.1_O_3−δ,_ Ir metal, and IrO_2_ by chronopotentiometry method. Regardless of potential fluctuations caused by intermittent O_2_ bubbles released from the electrode surface, fairly stable performance is observed for all samples. The stability for different catalysts is then compared using their S-numbers, which are calculated by dividing the amount of oxygen molecules evolved by the amount of Ir dissolved in the electrolyte^41^. Moreover, as the surface reconstruction with fast cation dissolution (Fig. 5a) may greatly affect the following S-number calculation, both SrCo_0.9_Ir_0.1_O_3−δ_ and Ir metal electrodes are pre-treated at 10 mA cm^−2^ for 60 min (Supplementary Figure 9) for reaching the steady state. Figure 5c shows S-numbers for SrCo_0.9_Ir_0.1_O_3−δ,_ Ir metal, and IrO_2_. Here, all S-numbers are calculated according to the amount of dissolved Ir measured during chronopotentiometry (Fig. 5b and Supplementary Figure 9). The S-numbers of different oxides reported by Geiger et al. are also presented^41^. Due to the initial surface reconstruction, much lower S-numbers (10^3^–10^4^) are observed from SrCo_0.9_Ir_0.1_O_3−δ_ and Ir metal during the pre-treatment stage (initial 60 min). Interestingly, from the following 180 min test, the S-number of SrCo_0.9_Ir_0.1_O_3−δ_ steeply increased to approximately 10^5^, which is approximately one order of magnitude higher than the S-numbers of reported perovskites. In addition, the S-numbers of SrCo_0.9_Ir_0.1_O_3−δ_ and Ir metal are comparable with the S-number of IrO_2_ (by Sigma-Aldrich). The S-number of IrO_2_ we measured, however, is much lower than that of reported IrO_2_ (by Alfa-Aesar)^41^. Such higher stability of reported IrO_2_ can be related to the improved stoichiometry on the surface after additional annealing step. It is likely the production or condition of raw materials may also affect the S-numbers. From these results, we can conclude that the eventually formed Ir-rich layer over SrCo_0.910_Ir_0.1_O_3−δ_ is rather stable if neglecting the initial instability caused by surface reconstruction process.

## Discussion

In summary, we developed a pseudo-cubic SrCo_0.9_Ir_0.1_O_3−δ_ perovskite. Such material can be easily prepared with a simple solid state method without high pressure condition. The Ir from pseudo-cubic SrCo_0.9_Ir_0.1_O_3−δ_ showed an intrinsic activity (TOF) more than two orders of magnitude higher than that in IrO_2_, and approximately 10 times higher than that in the benchmark PLD-SrIrO_3_. The chemical state of surface Ir in SrCo_0.9_Ir_0.1_O_3−δ_ is found changed after electrochemical cycling. The reconstructed surfaces are responsible for the observed activities. Although the initial bulk catalysts may not directly influence the OER, this study highlights that the chemical states of bulk catalysts, such as the crystal structure (pseudo-cubic structure with corner-shared IrO6 octahedrons) and the content of oxygen vacancy in the lattice (under-coordinated Ir), will affect the activity of the in-situ formed active sites (Ir-rich surface) for OER.

## Methods

### Synthesis of oxides

All composite oxides were synthesized with solid state method. Powders of SrCO_3_, IrO_2_, TiO_2_, and Co_3_O_4_ from Sigma-Aldrich Corporation were used as raw materials. Briefly, stoichiometry amount of raw materials were weighted and then mixed in mortar. The mixed precursors were finally calcined with box furnaces in ambient air. A sintering condition of 850 °C for 12 h, 1100 °C for 12° h, and 1200 °C for 12 h is applied for m-SrIrO_3_, SrCo_0.9_Ir_0.1_O_3−δ_, and SrCo_0.9_Ti_0.1_O_3−δ_ respectively.

### Characterizations

The phase structures of m-SrIrO_3_ and SrCo_0.9_Ir_0.1_O_3−δ_ were identified with X-ray Powder Diffractometer (D8 advance, Bruker Corporation). The phase structures were analyzed by the Rietveld method with GSAS program and EXPGUI interface^42^. The morphologies of the materials were studied by FESEM (JEOL 6340F). TEM images were collected on JEM-2010F (JEOL). The XPS tests were performed using a PHI-5400 equipment with Al Ka beam source (250 W) and position-sensitive detector. An XPSpeak41 software is applied for peak fitting. Considering the spin–orbit splitting, a relative area ratio of 2:3 and 3:4 is considered for the doublets in Sr_3*d* and in Ir_4*f*, respectively. A spin–orbit splitting of 3 eV is considered for the doublets in Ir_4*f*. A Shirley background was applied during the fitting. All peaks are described as convolution of Gaussian and Lorentzian function.

XAS experiments were performed at Singapore Synchrotron Light Source, XAFCA beamline. Data analysis were performed with the Athena software package. The surface areas were characterized by nitrogen adsorption–desorption tests (ASAP Tri-star II 3020) with the BET method.

### Electrochemical tests

The electrochemical experiments were carried out on a rotating electrode configuration from Pine Instrument at room temperature. The working electrode of glassy carbon (rotating disk) with an area of 0.196 cm^−2^ was applied. The catalyst ink with a concentration of 5 mg mL^−1^ was prepared by ultra-sonically dispersing 2.5 mg oxide and 1 mg acetylene black (Alfa Aesar) in an water (375 µL)–isopropanol (112.5 µL)–Nafion (12.5 µL) solution. 10 µL of well dispersed ink was dropped onto the glassy carbon and dried overnight. For ICP-MS tests, the amount of oxide increased to 5 mg for SrCo_0.9_Ir_0.1_O_3−δ_ and Ir metal. As a higher stability is expected for IrO_2_, its amount further increase to 10 mg. An SP-150 workstation (Bio-Logic Science Instruments) was applied to perform cyclic voltammetry scanning and chronopotentiometric tests. The tests were performed in a 0.1 M HClO_4_ solution, which was purged with ultra-pure oxygen before each measurement for approximately 30 min. A rotation speed of 1600 rpm was used. A Pt wire was used as the counter electrode and a saturated calomel electrode (SCE) was used as the reference electrode.

### TOF calculation

The TOF is calculated from the equation:

1 $${\mathrm{TOF}} = \frac{{j \times A_{\rm OX}}}{{4 \times e \times N_{\mathrm A}}}$$

where *j* is the BET surface area normalized current density at an overpotential of 270 mV. *A*_OX_ is the total surface area of the catalyst deposited on the GC electrode. *e* is the electric charge carried by a single electron. *N*_A_ is the number of Ir atoms. While calculating the *N*_A_, three cases are considered.

In the first case, only the Ir atoms from the outermost surface are considered. For PLD-SrIrO_3_, the calculation is performed by assuming PLD-SrIrO_3_ with an ideal P *m-3m* structure and a perfect (100) surface. A lattice parameter of 4.024 Å reported in the literature is used^13^. For m-SrIrO_3_, the lattice parameters from XRD refinement are used and a (001) surface, with the highest Ir surface density, is chosen (thus the activity will not be overestimated). For SrCo_0.9_Ir_0.1_O_3−δ_, the lattice parameters from XRD refinement are used and a (010) surface, with the highest Ir surface density, is chosen (thus the activity will not be overestimated). For IrO_2_, lattice parameters of *a* = 4.505 Å and *c* = 3.159 Å are used and a (110) surface is considered^43^. In this case, the active Ir atoms in reconstructed surface region are not counted and thus the activity is over-estimated. Thus, this way cannot be used for comparing TOF.

In the second case, all Ir atoms for each catalyst loaded on the electrode are considered active for OER. Please note this case over-estimates the active Ir atoms and thus the activity will be significantly under-estimated. Therefore, this way does not make sense, too.

In the third case, to include the effect of surface reconstruction, all Ir atoms from reconstructed surface regions are considered. Based on the TEM results (Fig. 2a, c), we assume the depth of the reconstructed surface region in m-SrIrO_3_ and SrCo_0.9_Ir_0.1_O_3−δ_ is 5 and 10 nm, respectively. In PLD-SrIrO_3_ film, it was reported that the root mean square roughness is <4 nm before OER and <20 nm after cycling^13^. Thus, we assume a surface reconstruction with a depth of 8 and 40 nm to normalize the initial activity and final activity (after 30 h test) of Ir in PLD-SrIrO_3_, respectively. For IrO_2_, the surface reconstruction effect is negligible, and thus is not considered.

### Estimation of the current due to Sr and Co leaching

Here, we estimate the maximum current contributed by Sr and Co leaching (possible leaching current). Several assumptions are made for the calculation/estimation:

1. The Sr^2+^ and Co^3+^ leached is compensated by electron lose from the catalyst, i.e., all leached Sr^2+^ and Co^3+^ cations will contribute to the current.
2. All Sr^2+^ and Co^3+^ from the surface region (10 nm depth, determined by TEM as shown in the manuscript) leached in 5 cycles at a constant speed. This is because we did not see steep current changes due to cation leaching.

Then, the current from cation leaching $\left( {{I}_{{\mathrm{ox}}}^{{\mathrm{leach}}}} \right)$ can be calculated by the equation:

2 $$I_{{\mathrm{ox}}}^{{\mathrm{leach}}} =\frac{{\left( {2 \times {n}_{{\mathrm{Sr}}^{2 + }} + 3 \times {n}_{{\mathrm{Co}}^{3 + }}} \right) \times {e}}}{{{t} \ast {S}_{{\mathrm{ox}}}}}$$

${n}_{{\mathrm{Sr}}^{2 + }}$ and ${n}_{{\mathrm{Co}}^{3 + }}$ are the number of leached Sr^2+^ and Co^3+^ from the surface region (10 nm depth). *e* is the electric charge of an electron. *t* is the time used for 5 cycles. Our cycling test for SrCo_0.9_Ir_0.1_O_3−δ_ is performed in the potential range (vs. RHE) from 1 to 1.7 V. The scan rate is 10 mV s^−1^. *S*_*ox*_ is the surface area of the loaded catalyst.

Based on that, a maximum current of 0.0186 ${\mathrm{mA}}\;{\mathrm{cm}}_{{\mathrm{ox}}}^{ - 2}$ is calculated, and which can contribute to the measured current in initial 5 cycles. This current value is more than two orders of magnitude lower than the measured OER current from SrCo_0.9_Ir_0.1_O_3−δ_. For example, at an overpotential of 270 and 320 mV, the measured OER current from SrCo_0.9_Ir_0.1_O_3−δ_ can reach 2.8 and 16.3 ${\mathrm{mA}}\;{\mathrm{cm}}_{{\mathrm{ox}}}^{ - 2}$, respectively.

### DFT calculation

Spin-polarized DFT calculations were performed using the Vienna ab initio simulation package (VASP)^44,45^ with the projector-augmented wave (PAW) approach^46^ and the Perdew–Burk–Ernzerhof (PBE) exchange-correlation functional^47^. To account for the strongly localized *d*-electrons of Co, a DFT+U approach was adopted and an effective Hubbard U parameter of 3.32 eV was used^48,49^. The electronic energy tolerance was set to 10^−6^ eV and the force tolerance for structural relaxation was 0.015 eV Å^−1^. The structural model for PLD-SrIrO_3_ is a 2 × 2 × 2 cubic supercell, for m-SrIrO_3_ is a conventional standard unit cell and for pc-SrCo_0.875_Ir_0.125_O_3_ is a 2 × 1 × 1 orthorhombic supercell. For all compositions, the lattice constants and ion positions were first fully relaxed. Then, the defect calculations were performed on the fully relaxed stoichiometric structures, and all the symmetry distinct oxygen sites in the corresponding model were explored to search for the most stable vacancy site. The oxygen vacancy concentration for PLD-SrIrO_3_ and pc-SrCo_0.875_Ir_0.125_O_3_ is 4.167% while for m-SrIrO_3_ is 3.125%. The total energy calculations of all structures were performed with the tetrahedron method with Blöchl corrections^50^ and an energy cutoff of 520 eV. And a 6 × 6 × 6 Monkhorst–Pack k-point mesh was employed for PLD-SrIrO_3_, a 4 × 6 × 8 mesh for pc-SrCo_0.875_Ir_0.125_O_3_ and a 10 × 6 × 4 mesh for m-SrIrO_3_. For all compositions, the bulk oxygen vacancy formation energy was calculated based on the most stable oxygen-deficient structure and with respect to H_2_O (g) and H_2_ (g) at standard condition. The oxygen vacancy formation enthalpy will be shifted around +2.33 eV larger if O_2_ (g) is used as the reference.

## Supplementary information

Supplementary Information

## Data Availability

All relevant data are available from the authors upon request.

## References

[1] Ginley D, Green MA, Collins R (2008). Solar energy conversion toward 1 terawatt. MRS Bull..

[2] Strmcnik D (2013). Improving the hydrogen oxidation reaction rate by promotion of hydroxyl adsorption. Nat. Chem..

[3] Danilovic N (2012). Enhancing the alkaline hydrogen evolution reaction activity through the bifunctionality of Ni(OH)_2_/metal catalysts. Angew. Chem. Int. Ed..

[4] Reier T, Oezaslan M, Strasser P (2012). Electrocatalytic oxygen evolution reaction (OER) on Ru, Ir, and Pt catalysts: a comparative study of nanoparticles and bulk materials. ACS Catal..

[5] Antolini E (2014). Iridium as catalyst and cocatalyst for oxygen evolution/reduction in acidic polymer electrolyte membrane electrolyzers and fuel cells. ACS Catal..

[6] Zhao YX, Hernandez-Pagan EA, Vargas-Barbosa NM, Dysart JL, Mallouk TE (2011). A high yield synthesis of ligand-free iridium oxide nanoparticles with high electrocatalytic activity. J. Phys. Chem. Lett..

[7] Lee Y, Suntivich J, May KJ, Perry EE, Shao-Horn Y (2012). Synthesis and activities of rutile IrO_2_ and RuO_2_ nanoparticles for oxygen evolution in acid and alkaline solutions. J. Phys. Chem. Lett..

[8] Lim J (2018). Ultrathin IrO_2_ nanoneedles for electrochemical water oxidation. Adv. Funct. Mater..

[9] Pi YC, Zhang N, Guo SJ, Guo J, Huang XQ (2016). Ultrathin laminar Ir superstructure as highly efficient oxygen evolution electrocatalyst in broad pH range. Nano Lett..

[10] Oh HS, Nong HN, Reier T, Gliech M, Strasser P (2015). Oxide-supported Ir nanodendrites with high activity and durability for the oxygen evolution reaction in acid PEM water electrolyzers. Chem. Sci..

[11] Nong HN (2015). Oxide-supported IrNiO_*x*_ core–shell particles as efficient, cost-effective, and stable catalysts for electrochemical water splitting. Angew. Chem. Int. Ed..

[12] Diaz-Morales O (2016). Iridium-based double perovskites for efficient water oxidation in acid media. Nat. Commun..

[13] Seitz LC (2016). A highly active and stable IrO_*x*_/SrIrO_3_ catalyst for the oxygen evolution reaction. Science.

[14] Tang RB (2016). Oxygen evolution reaction electrocatalysis on SrIrO_3_ grown using molecular beam epitaxy. J. Mater. Chem. A.

[15] Grimaud A (2017). Activation of surface oxygen sites on an iridium-based model catalyst for the oxygen evolution reaction. Nat. Energy.

[16] Liu YX, Masumoto H, Goto T (2005). Structural, electrical and optical characterization of SrIrO_3_ thin films prepared by laser-ablation. Mater. Trans..

[17] Zhao JG (2008). High-pressure synthesis of orthorhombic SrIrO_3_ perovskite and its positive magnetoresistance. J. Appl. Phys..

[18] Longo JM, Kafalas JA, Arnott RJ (1971). Structure and properties of high and low pressure forms of SrIrO_3_. J. Solid State Chem..

[19] Qasim I, Kennedy BJ, Avdeev M (2013). Synthesis, structures and properties of transition metal doped SrIrO_3_. J. Mater. Chem. A.

[20] Sun SN, Li HY, Xu ZCJ (2018). Impact of surface area in evaluation of catalyst activity. Joule.

[21] Danilovic N (2014). Using surface segregation to design stable Ru–Ir oxides for the oxygen evolution reaction in acidic environments. Angew. Chem. Int. Ed..

[22] Massue C (2017). Microwave-assisted synthesis of stable and highly active Ir oxohydroxides for electrochemical oxidation of water. ChemSusChem.

[23] Willinger E, Massue C, Schlogl R, Willinger MG (2017). Identifying key structural features of IrO_*x*_ water splitting catalysts. J. Am. Chem. Soc..

[24] Mutoro E, Crumlin EJ, Biegalski MD, Christen HM, Shao-Horn Y (2011). Enhanced oxygen reduction activity on surface-decorated perovskite thin films for solid oxide fuel cells. Energy Environ. Sci..

[25] Vasquez RP (1991). X-ray photoelectron-spectroscopy study of Sr and Ba compounds. J. Electron Spectrosc..

[26] Wertheim GK, Guggenheim HJ (1980). Conduction-electron screening in metallic oxides—IrO_2_. Phys. Rev. B.

[27] Pfeifer V (2016). The electronic structure of iridium oxide electrodes active in water splitting. Phys. Chem. Chem. Phys..

[28] Atanasoska L, Atanasoski R, Trasatti S (1990). XPS and AES study of mixed layers of RuO_2_ and IrO_2_. Vacuum.

[29] Kodintsev IM, Trasatti S, Rubel M, Wieckowski A, Kaufher N (1992). X-ray photoelectron-spectroscopy and electrochemical surface characterization of IrO_2_ + RuO_2_ electrodes. Langmuir.

[30] Peuckert M (1984). XPS study on thermally and electrochemically prepared oxidic adlayers on iridium. Surf. Sci..

[31] Minguzzi A (2015). Easy accommodation of different oxidation states in iridium oxide nanoparticles with different hydration degree as water oxidation electrocatalysts. ACS Catal..

[32] Smith RDL, Sporinova B, Fagan RD, Trudel S, Berlinguette CP (2014). Facile photochemical preparation of amorphous iridium oxide films for water oxidation catalysis. Chem. Mater..

[33] Pfeifer V (2016). The electronic structure of iridium and its oxides. Surf. Interface Anal..

[34] Li T (2018). Atomic-scale insights into surface species of electrocatalysts in three dimensions. Nat. Catal..

[35] Minguzzi A (2014). Observing the oxidation state turnover in heterogeneous iridium-based water oxidation catalysts. Chem. Sci..

[36] Wang HY (2016). In operando identification of geometrical-site-dependent water oxidation activity of spinel Co_3_O_4_. J. Am. Chem. Soc..

[37] Mefford JT (2016). Water electrolysis on La_1−*x*_Sr_*x*_CoO_3-delta_ perovskite electrocatalysts. Nat. Commun..

[38] Lee YL, Kleis J, Rossmeisl J, Shao-Horn Y, Morgan D (2011). Prediction of solid oxide fuel cell cathode activity with first-principles descriptors. Energy Environ. Sci..

[39] Tejuca LG, Bell AT, Fierro JLG, Pena MA (1988). Surface behavior of reduced LaCoO_3_ as studied by TPD of CO, CO_2_ and H_2_ probes and by XPS. Appl. Surf. Sci..

[40] Pishahang M, Bakken E, Stolen S, Larring Y, Thomas CI (2013). Oxygen non-stoichiometry and redox thermodynamics of LaMn_1−*x*_Co_*x*_O_3-delta_. Solid State Ion..

[41] Geiger S (2018). The stability number as a metric for electrocatalyst stability benchmarking. Nat. Catal..

[42] Toby BH (2001). EXPGUI, a graphical user interface for GSAS. J. Appl. Crystallogr..

[43] Sen FG (2015). Towards accurate prediction of catalytic activity in IrO_2_ nanoclusters via first principles-based variable charge force field. J. Mater. Chem. A.

[44] Kresse G, Furthmuller J (1996). Efficient iterative schemes for ab initio total-energy calculations using a plane-wave basis set. Phys. Rev. B.

[45] Kresse G, Furthmuller J (1996). Efficiency of ab-initio total energy calculations for metals and semiconductors using a plane-wave basis set. Comput. Mater. Sci..

[46] Blochl PE (1994). Projector augmented-wave method. Phys. Rev. B.

[47] Perdew JP, Burke K, Ernzerhof M (1996). Generalized gradient approximation made simple. Phys. Rev. Lett..

[48] Wang L, Maxisch T, Ceder G (2006). Oxidation energies of transition metal oxides within the GGA+U framework. Phys. Rev. B.

[49] Lee YL, Kleis J, Rossmeisl J, Morgan D (2009). Ab initio energetics of LaBO_3_(001) (B = Mn, Fe, Co, and Ni) for solid oxide fuel cell cathodes. Phys. Rev. B.

[50] Blochl PE, Jepsen O, Andersen OK (1994). Improved tetrahedron method for Brillouin-zone integrations. Phys. Rev. B.

