# Supplementary material for: Exceptionally active iridium evolved from a pseudo-cubic perovskite for oxygen evolution in acid
Source: Nat Commun. 2019 Feb 4;10:572. doi: 10.1038/s41467-019-08532-3 (PMC6362036; doi:10.1038/s41467-019-08532-3)
Supplement: Supplementary file 1 — Supplementary Information [file 41467_2019_8532_MOESM1_ESM.pdf]

Supplementary information for

Exceptionally active iridium evolved from a pseudo-cubic perovskite for oxygen evolution in acid

Chen et al.

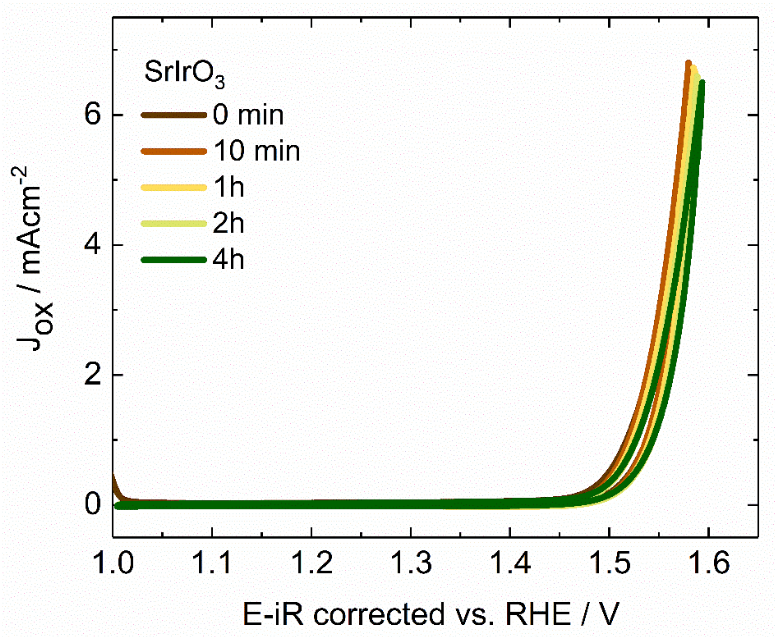

Supplementary Figure 1. CV of m-$SrIrO_3$. The CV measurements from m-$SrIrO_3$ during a 4h chronopotentiometric test at 10mA cm^{-2}_{GEO}. The CV is recorded at 10 mV s^{-1} after pausing the chronopotentiometric test, and the current is normalized to BET surface area.

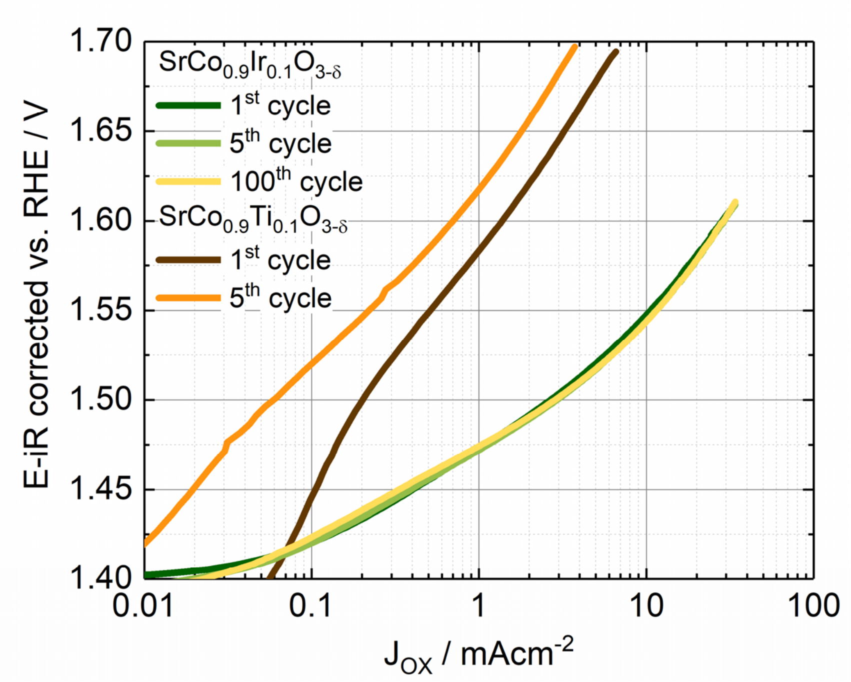

Supplementary Figure 2. Specific activity comparison in 5 cycles. Tafel plots of the specific activities of $SrCo_{0.9}Ir_{0.1}O_{3-\delta}$ and $SrCo_{0.9}Ti_{0.1}O_{3-\delta}$ in 0.1 M $HClO_4$. The $SrCo_{0.9}Ti_{0.1}O_{3-\delta}$ is a perovskite with cubic structure and is reported with good activity for OER in alkaline electrolyte.[1] The BET area for $SrCo_{0.9}Ti_{0.1}O_{3-\delta}$ is 0.100 m^2g^{-1}. Here, firstly, the initial specific activity of $SrCo_{0.9}Ti_{0.1}O_{3-\delta}$ is one to two orders of magnitude lower than that of $SrCo_{0.9}Ir_{0.1}O_{3-\delta}$. Secondly, within 5 cycles, the activity of $SrCo_{0.9}Ti_{0.1}O_{3-\delta}$ fast degraded while the $SrCo_{0.9}Ir_{0.1}O_{3-\delta}$ maintained its activity. These two results confirmed Co itself in $SrCo_{0.9}Ir_{0.1}O_{3-\delta}$ has little contribute to the observed activity of $SrCo_{0.9}Ir_{0.1}O_{3-\delta}$. Additionally, as discussed in the following sections, a rapid dissolution of Sr and Co is expected in acidic electrolytes. The much lower current densities from $SrCo_{0.9}Ti_{0.1}O_{3-\delta}$ also indicate that the leaching of Sr and Co won't give a "fake" high OER current. It is indeed the Ir-rich surface on $SrCo_{0.9}Ir_{0.1}O_{3-\delta}$ that gives the high OER activity. In addition, an estimated maximum current due to Sr and Co leaching in initial 5 cycles is more than two orders of magnitude lower than the measured OER current from $SrCo_{0.9}Ir_{0.1}O_{3-\delta}$. (see the estimation of the current due to Sr and Co leaching in the **Methods**)

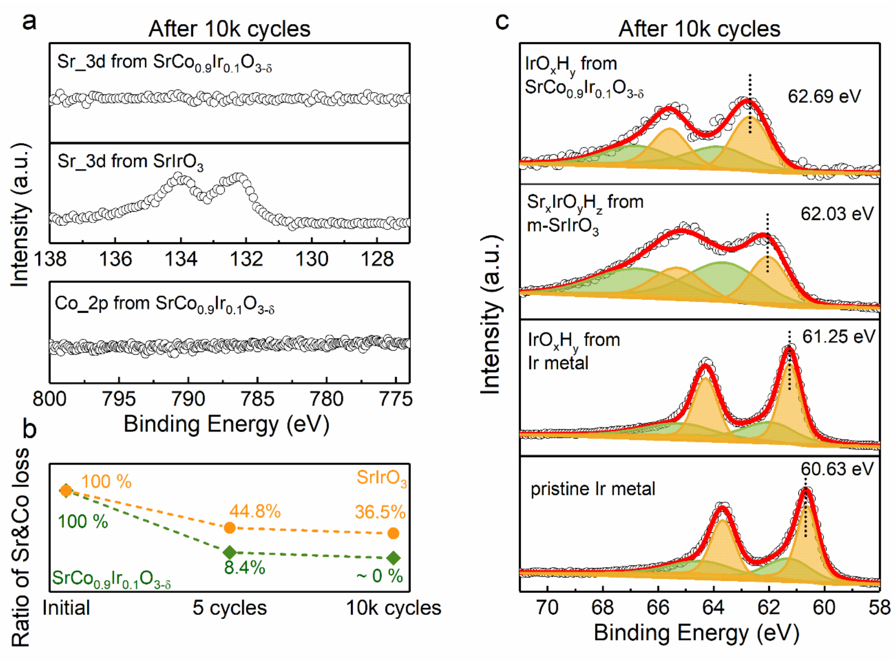

Supplementary Figure 3. XPS characterization. a, Sr_3d and Co_2p XPS from m-$SrIrO_3$ and $SrCo_{0.9}Ir_{0.1}O_{3-\delta}$ after 10k CV cycles. **b**, the ratio of residual Sr and Co in the surface of m-$SrIrO_3$ and $SrCo_{0.9}Ir_{0.1}O_{3-\delta}$ after 10k CV cycles. **c**, Ir_4f XPS from Ir, m-$SrIrO_3$, and $SrCo_{0.9}Ir_{0.1}O_{3-\delta}$ after 10k CV cycles. The signal from pristine Ir metal is also presented for comparison. XPS fit parameters for Ir_4f of all samples are shown in **Supplementary Table 5**.

As shown in **Supplementary Figure 3a**, after 10k cycles, the content of residual Sr and Co from the surface region of $SrCo_{0.9}Ir_{0.1}O_{3-\delta}$ is below the detection limitation of XPS. As summarized in **Supplementary Figure 3b**, a high amount of Sr (36.5 %) remains in the surface region of m-$SrIrO_3$ after 10k cycles. The spectra of Ir_4f from different samples are shown in **Supplementary Figure 3c**. The Ir_4f signal from Ir metal is also measured for comparison. By 10k cycles, the Ir_4f 7/2 peak shifts from 60.63 eV to 61.25 eV. This binding energy shift over Ir metal is similar to what has been reported in previous studies focusing on the formation of hydrous IrO_x on metallic Ir surface.[2, 3] From m-$SrIrO_3$ after 10k cycles, a binding energy of 62.03 eV of Ir_4f 7/2 peak is observed. An even higher binding energy of 62.69 eV of Ir_4f 7/2 peak is observed from $SrCo_{0.9}Ir_{0.1}O_{3-\delta}$ after 10k cycles. Such high binding energy indicates the hydrous IrO_x formed on $SrCo_{0.9}Ir_{0.1}O_{3-\delta}$ is different from those formed on m-$SrIrO_3$ and Ir metal.

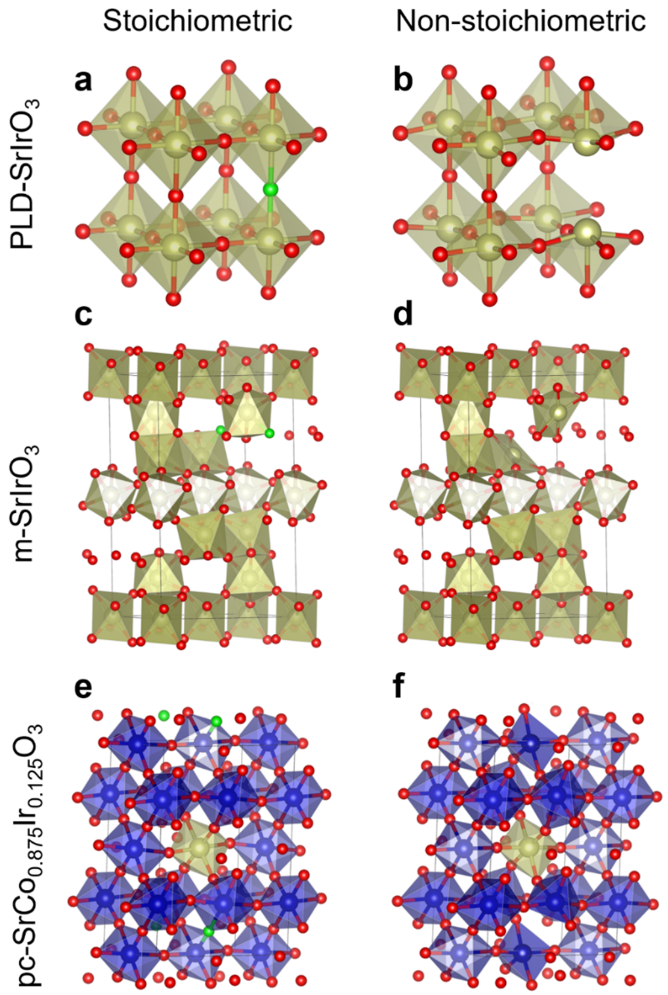

Supplementary Figure 4. Crystal structural models. The structural models for stoichiometric and non-stoichiometric PLD-$SrIrO_3$ (**a&b**), m-$SrIrO_3$ (**c&d**) and pc-$SrCo_{0.875}Ir_{0.125}O_3$ (**e&f**). The spheres in red, golden brown and blue colour represent O, Ir, and Co. The oxygen at the oxygen vacancy site is marked as green sphere. The Sr is not shown in all structural models. The non-stoichiometric phase for each compound is obtained after a comprehensive search for the most stable oxygen-deficient configuration.

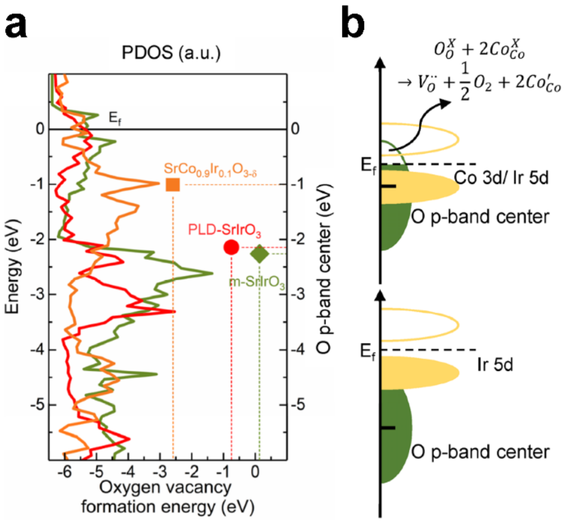

Supplementary Figure 5. PDOS characterization. a, Calculated projected density of states (PDOS) of O 2p, O p-band centers, and oxygen vacancy formation energies from m-$SrIrO_3$, PLD-$SrIrO_3$ and $SrCo_{0.9}Ir_{0.1}O_{3-\delta}$. The calculated O p-band center from $SrCo_{0.9}Ir_{0.1}O_3$ (-1.0 eV) is very close to the Fermi level. Instead, the O p-band centers from PLD-$SrIrO_3$ (-2.14 eV) and m-$SrIrO_3$ (-2.26 eV) are far away from the Fermi level. From a high vacancy formation energy of 0.14 eV, the formation of oxygen vacancies in m-$SrIrO_3$ is expected to be more difficult than $SrCo_{0.9}Ir_{0.1}O_3$. The PLD-$SrIrO_3$ is in pseudo-cubic structure and shows a relatively low vacancy formation energy of -0.75 eV, indicating the pseudo-cubic structure can facilitate the formation of oxygen vacancies. Nevertheless, a high amount of oxygen vacancies is expected in the $SrCo_{0.9}Ir_{0.1}O_3$ due to a much low vacancy formation energy of -2.59 eV. **b**, Schematic of band structures for PLD-/m-$SrIrO_3$ and $SrCo_{0.9}Ir_{0.1}O_3$. The formation of oxygen vacancies with the release of O_2 is presented using Kroger-Vink notation. A strong hybridization between O 2p band and Co 3d/Ir 5d in the $SrCo_{0.9}Ir_{0.1}O_3$ induces the release of lattice oxygen for the formation of oxygen vacancies.

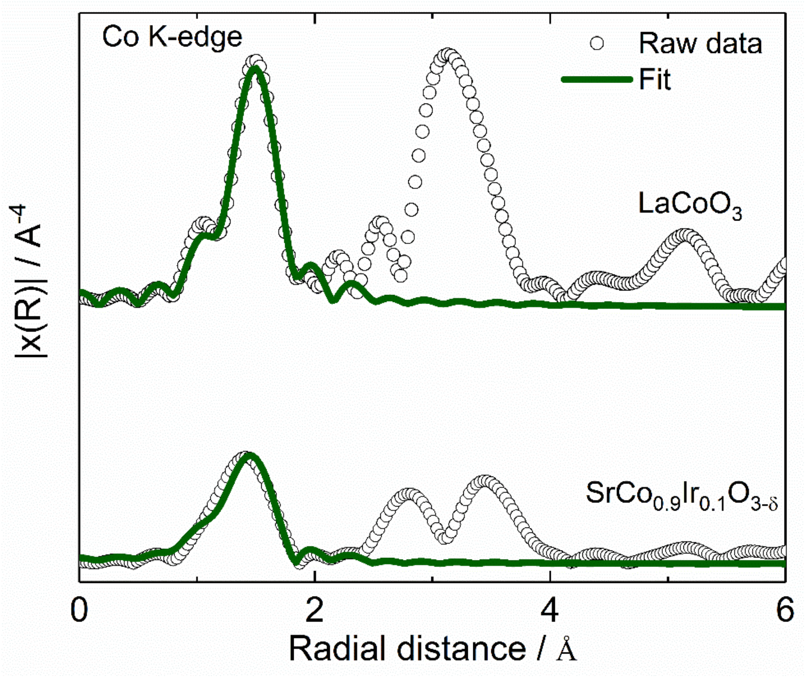

Supplementary Figure 6. EXAFS fitting. Fourier transformed k3-weighted Co K edge EXAFS recorded from $LaCoO_3$ and $SrCo_{0.9}Ir_{0.1}O_{3-\delta}$. A one-shell model was used to fit the first peak in the FT EXAFS spectra. The fitting ranges in k-space and R-space are 2-12 Å $^{-1}$ and 1-2 Å, respectively. Detailed results are in **Supplementary Table 7**.

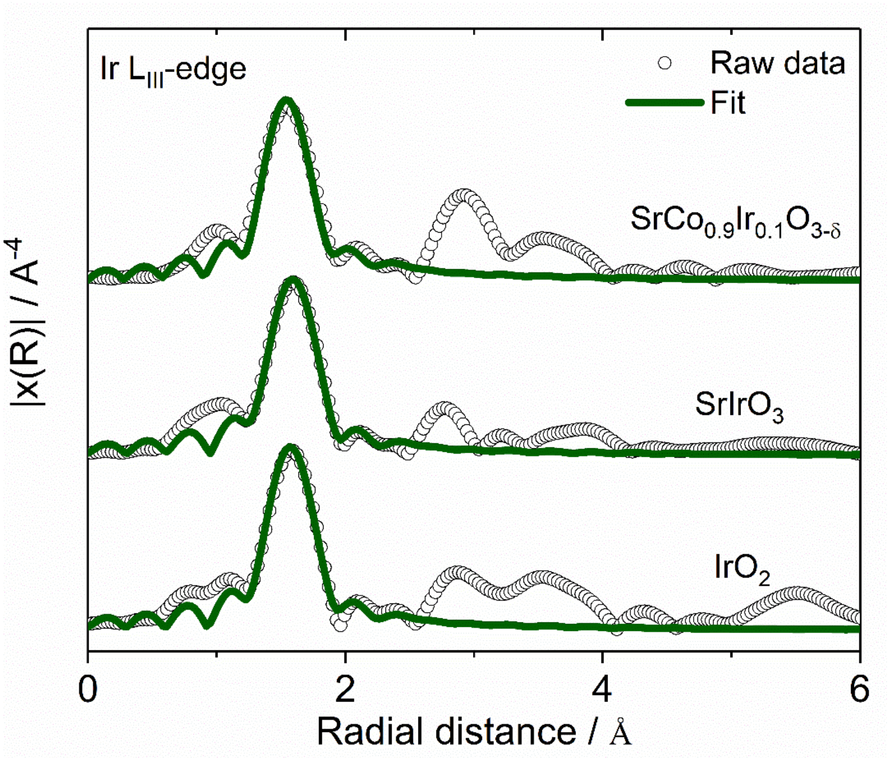

Supplementary Figure 7. EXAFS fitting. Fourier transformed k3-weighted Ir L_{III}-edge EXAFS recorded from $SrCo_{0.9}Ir_{0.1}O_{3-\delta}$, $SrIrO_3$ and IrO_2. A one-shell model was used to fit the first peak in the FT EXAFS spectra. The fitting ranges in k-space and R-space are 2-12 $Å^{-1}$ and 1-2 Å, respectively. Detailed results are in **Supplementary Table 8**.

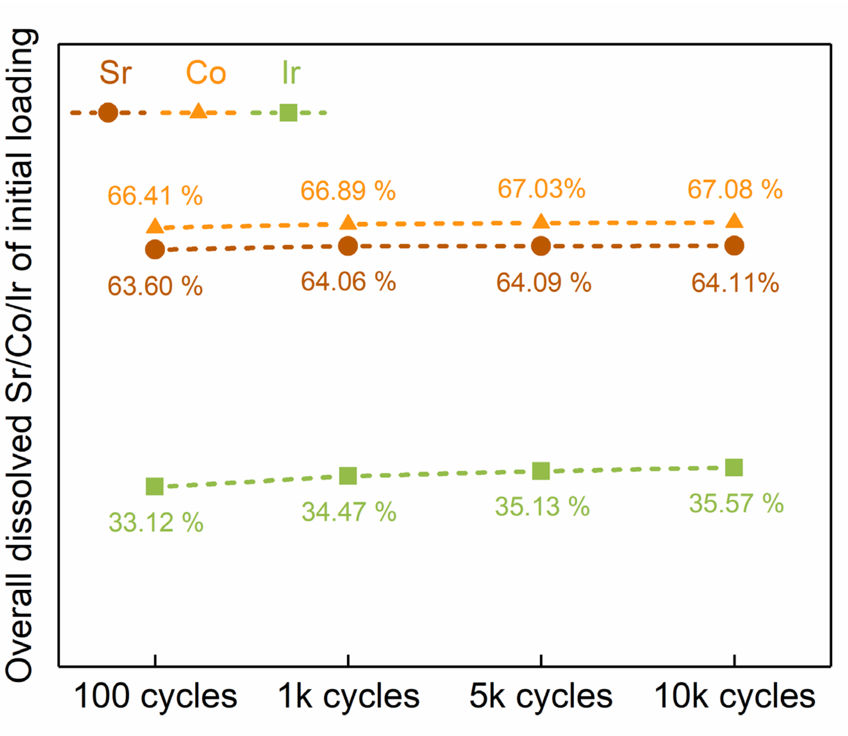

Supplementary Figure 8. Cation dissolution during CV. Overall dissolved Sr, Co, and Ir as % of initial loading during the CV cycling test from **Figure 5a**. At the first 100 cycles, a mass loss of 63.60 %, 66.41 %, and 33.12 % is found for Sr, Co, and Ir, respectively. This is due to the initial surface reconstruction process. Nevertheless, in the following 9.9k cycles, only tiny amounts of Sr, Co, and Ir are dissolved from $SrCo_{0.9}Ir_{0.1}O_{3-\delta}$.

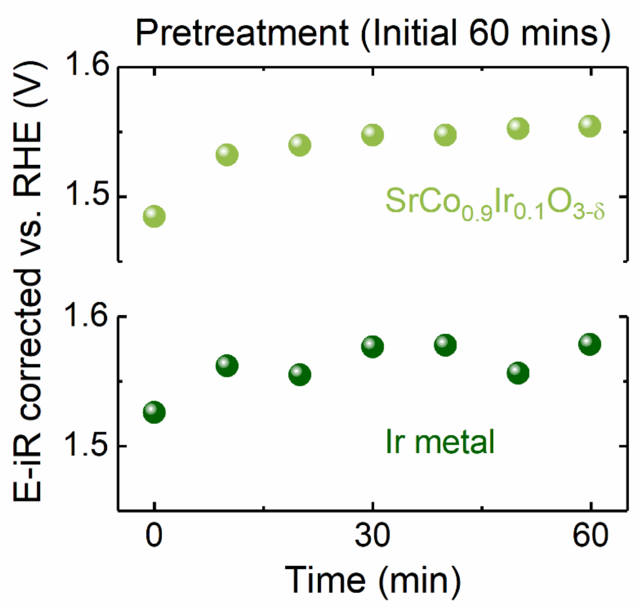

Supplementary Figure 9. Pre-treatment by chronopotentiometry. The pre-treatment (initial 60 min) by chronopotentiometry at 10 mA cm^{-2} for $SrCo_{0.9}Ir_{0.1}O_{3-\delta}$ and Ir metal.

Supplementary Table 1. Refined structure information

	$SrCo_{0.9}Ir_{0.1}O_{3-\delta}$		
Space group		P *nma*	
a(Å)		5.4626(5)	
b(Å)		7.7716(1)	
c(Å)		5.4634(5)	
		Sr	
Wyckoff		4c	
site		(0.0048(12), 1/4, -0.0049(38))	
Occ.		1	
U_{iso} (Å^2)		0.0273	
	Co		Ir
Wyckoff		4b	
site		(0, 0, 1/2)	
Occ.	0.907(0.003)		0.093(0.003)
U_{iso} (Å^2)		0.0326	
		O1	
Wyckoff		4c	
site		(0.0682(57), 1/4, 0.493(13))	
Occ.		1*	
U_{iso} (Å^2)		0.0661	
		O2	
Wyckoff		8d	
site		(0.200(2), -0.0070(63), 0.2549(31))	
Occ.		1*	
U_{iso} (Å^2)		0.0871	

*The occupancy of oxygen was fixed during the refinement.

Supplementary Table 2. XPS fit parameters for Sr_3d and Ir_4f of m-$SrIrO_3$

	**Pristine $SrIrO_3$**				**5 times cycled m-$SrIrO_3$**			
				**Sr**				
	$3d_{3/2}$	$3d_{5/2}$	$3d_{3/2}$	$3d_{5/2}$	$3d_{3/2}$	$3d_{5/2}$	$3d_{3/2}$	$3d_{5/2}$
**Binding energy (eV)**	132.3	130.55	133.35	131.81	132.88	131.15	134.49	132.62
**FWHM (eV)**	1.09	1	2.69	2.42	0.97	1.08	3.51	2.77
				**Ir**				
	$4f_{5/2}$	$4f_{7/2}$	$4f_{5/2}$	$4f_{7/2}$	$4f_{5/2}$	$4f_{7/2}$	$4f_{5/2}$	$4f_{7/2}$
**Binding energy (eV)**	63.33	60.33	64.7	61.7	64.19	61.19	66.35	63.35
**FWHM (eV)**	1.87	1.57	2.96	3.15	2.59	1.98	4.19	3.99

Supplementary Table 3. XPS fit parameters for Sr_3d and Ir_4f of $SrCo_{0.9}Ir_{0.1}O_{3-\delta}$.

	**Pristine $SrCo_{0.9}Ir_{0.1}O_{3-\delta}$**				**5 times cycled $SrCo_{0.9}Ir_{0.1}O_{3-\delta}$**			
				**Sr**				
			$3d_{3/2}$	$3d_{5/2}$				
**Binding energy (eV)**	/		133.6	131.83	/			
**FWHM (eV)**			2.42	2.38				
				**Ir**				
	$4f_{5/2}$*	$4f_{7/2}$	$4f_{5/2}$	$4f_{7/2}$	$4f_{5/2}$	$4f_{7/2}$	$4f_{5/2}$	$4f_{7/2}$
**Binding energy (eV)**	62.7	59.7	64.98	61.98	63.98	60.98	65.53	62.53
**FWHM (eV)**	2.84	2.84	2.79	2.79	2.33	1.75	4.14	4.29

*The FWMH for the doublet from Ir_4f in Pristine $SrCo_{0.9}Ir_{0.1}O_{3-\delta}$ is constrained to be equal.

Supplementary Table 4. XPS fit parameters for Ir_4f of IrO_2.

		$4f_{5/2}$	$4f_{7/2}$	$4f_{5/2}$ sat_1	$4f_{5/2}$ sat_2	$4f_{7/2}$ sat_1
**Pristine IrO_2**	**Binding energy (eV)**	64.59	61.61	65.56	67.6	62.57
	**FWHM (eV)**	1.11	0.92	2.19	2.01	2.41
**Cycled IrO_2**	**Binding energy (eV)**	64.61	61.63	65.49	67.76	62.51
	**FWHM (eV)**	1.06	0.95	2.49	2.31	2.76

Supplementary Table 5. XPS fit parameters for Ir_4f of pristine Ir and 10k times cycled Ir, m-$SrIrO_3$, and $SrCo_{0.9}Ir_{0.1}O_{3-\delta}$.

	**Pristine Ir**				**10k times cycled m-$SrIrO_3$**			
	$4f_{5/2}$	$4f_{7/2}$	$4f_{5/2}$	$4f_{7/2}$	$4f_{5/2}$	$4f_{7/2}$	$4f_{5/2}$	$4f_{7/2}$
**Binding energy (eV)**	63.67	60.63	64.41	61.28	65.28	62.03	66.71	63.63
**FWHM (eV)**	1.04	0.88	2.77	2.03	2.13	1.63	3.72	3.15
	**10k times cycled Ir**				**10k times cycled $SrCo_{0.9}Ir_{0.1}O_{3-\delta}$**			
	$4f_{5/2}$	$4f_{7/2}$	$4f_{5/2}$	$4f_{7/2}$	$4f_{5/2}$	$4f_{7/2}$	$4f_{5/2}$	$4f_{7/2}$
**Binding energy (eV)**	64.28	61.25	65.21	61.97	65.58	62.69	66.8	63.84
**FWHM (eV)**	1.02	0.91	3.1	2.45	1.55	1.59	2.84	2.84

Supplementary Table 6. Oxygen vacancy formation enthalpy

Sample	O vacancy percent (%)	Oxygen vacancy formation enthalpy (eV)
PLD-$SrIrO_3$	4.167	-0.74589
m-$SrIrO_3$	3.125	0.14067
$SrCo_{0.9}Ir_{0.1}O_{3-\delta}$	4.167	-2.58998

Supplementary Table 7. Fitting parameters of the Fourier-transformed k^3-weighted Co K-edge EXAFS from $LaCoO_3$ and $SrCo_{0.9}Ir_{0.1}O_{3-\delta}$.

	**Co-O (Å)**	**CN[1]**	**σ^2 (Å^2)[2]**	**ΔE_0[3]**
$LaCoO_3$	1.927(0.009)	6.0(0.5)	0.0031(0.0012)	0.19(0.96)
$SrCo_{0.9}Ir_{0.1}O_{3-\delta}$	1.909(0.012)	4.1(0.5)	0.0070(0.0019)	-0.77(1.22)

1. **CN:** Coordination number
2. **σ^2 :** Mean-square-displacement in R
3. **ΔE_0**: Energy shift

Supplementary Table 8. Fit parameters of the Fourier-transformed k3-weighted Ir L_{III}-edge EXAFS from IrO_2, m-$SrIrO_3$ and $SrCo_{0.9}Ir_{0.1}O_{3-\delta}$.

	**Ir-O (Å)**	**CN**	**σ^2 (Å^2)**	**ΔE_0**
IrO_2	1.970(0.019)	6.0(1.0)	0.0016(0.0024)	12.6(2.2)
m-$SrIrO_3$	1.989(0.020)	6.3(1.2)	0.0022(0.0026)	13.4(2.3)
$SrCo_{0.9}Ir_{0.1}O_{3-\delta}$	1.933(0.019)	4.9(0.9)	0.0006(0.0023)	12.5(2.3)

Supplementary References

1. Su C, *et al.* $SrCo_{0.9}Ti_{0.1}O_{3-delta}$ as a new electrocatalyst for the oxygen evolution reaction in alkaline electrolyte with stable performance. *Acs Appl. Mater. Inter.* **7**, 17663-17670 (2015).

2. Li T, *et al.* Atomic-scale insights into surface species of electrocatalysts in three dimensions. *Nat. Catal.* **1**, 300-305 (2018).

3. Pfeifer V, *et al.* The electronic structure of iridium oxide electrodes active in water splitting. *Phys Chem Chem Phys* **18**, 2292-2296 (2016).
